# The ideal mHealth-application for rheumatoid arthritis: qualitative findings from stakeholder focus groups

**DOI:** 10.1186/s12891-021-04624-8

**Published:** 2021-08-30

**Authors:** Michaël Doumen, René Westhovens, Sofia Pazmino, Delphine Bertrand, Veerle Stouten, Claudia Neys, Nelly Creten, Els Van Laeken, Patrick Verschueren, Diederik De Cock

**Affiliations:** 1 Skeletal Biology and Engineering Research Centre, KU Leuven, Leuven, Belgium; 2 Rheumatology, University Hospitals Leuven, Leuven, Belgium; 3 Patient Experts Rheumatology, ReumaNet, Leuven, Belgium

**Keywords:** Rheumatoid arthritis, eHealth, mHealth, Mobile apps, Stakeholders, Perceptions, Focus groups, Qualitative, Care innovations

## Abstract

**Background:**

Shifts in treatment strategies for rheumatoid arthritis (RA) have made ambulatory care more labour-intensive. These developments have prompted innovative care models, including mobile health (mHealth) applications. This study aimed to explore the perceptions of mHealth-inexperienced stakeholders concerning these applications in RA care.

**Methods:**

We performed a qualitative study by focus group interviews of stakeholders including RA patients, nurses specialised in RA care and rheumatologists. The qualitative analysis guide of Leuven (QUAGOL), which is based on grounded theory principles, was used to thematically analyse the data. In addition, the Persuasive Systems Design (PSD) model was used to structure recommended app-features.

**Results:**

In total, 2 focus groups with nurses (total *n* = 16), 2 with patients (*n* = 17) and 2 with rheumatologists (*n* = 25) took place. Six overarching themes emerged from the analysis. Efficiency of care and enabling patient empowerment were the two themes considered as expected benefits of mHealth-use in practice by the stakeholders. In contrast, 4 themes emerged as possible barriers of mHealth-use: the burden of chronic app-use, motivational aspects, target group aspects, and legal and organisational requirements. Additionally, recommendations for an ideal mHealth-app could be structured into 4 domains (Primary Task Support, Dialogue Support, Social Support and System Credibility) according to the PSD-framework. Most recommended features were related to improving ease of use (Task Support) and System Credibility.

**Conclusions:**

Although mHealth-apps were expected to improve care efficiency and stimulate patient empowerment, stakeholders were concerned that mHealth-app use could reinforce negative illness behaviour. For mHealth-apps to be successful in practice, challenges according to stakeholders were avoiding long-term poor compliance, finding the target audience and tailoring a legal and organisational framework. Finally, the ideal mHealth-application should above all be trustworthy and easy to use.

**Supplementary Information:**

The online version contains supplementary material available at 10.1186/s12891-021-04624-8.

## Background

Rheumatoid arthritis (RA) is a chronic inflammatory autoimmune condition that primarily results in joint pain and swelling as well as functional disability and impaired quality of life [1]. Current recommendations for the management of RA state that treatment should be started early and intensively to a target of remission or low disease activity (LDA) [2]. This treat-to-target (T2T) strategy and novel disease-modifying antirheumatic drugs (DMARDs) have considerably improved clinical outcomes in RA. However, in order to achieve optimal outcomes, pharmacological treatment should be started within a certain window of opportunity [3], while specific additional patient needs should simultaneously be addressed [4]. Nonetheless, early access to specialised rheumatological care is not always readily available [5]. In part due to the introduction of more labour-intensive T2T-strategies and person-centred care, some countries face a relative shortage of practicing rheumatologists [6]. Additionally, in many countries, the role of other healthcare professionals, including nurse-specialists, is not firmly established in care trajectories for patients with RA [7]. Therefore, attention is turning towards new care models for RA, including the use of mobile health (mHealth) applications, such as mobile apps and wearables.

In recent years, an ongoing evolution in technology and digitalisation has resulted in an ever-increasing number of mHealth-applications [8]. In addition, wearable activity trackers (WATs) are emerging as a promising tool to monitor biometric data [9]. When routinely integrated into health care and linked to electronic medical patient files, these mHealth-applications and WATs could prove to be an added value in (tele)monitoring treatment adherence, adverse treatment effects or symptoms and disease activity in RA [10]. Specifically, remote monitoring of a well-selected set of patient-reported outcomes (PROs) and wearable-obtained physical parameters could provide continuous information on a patient’s health status [11]. Since the T2T-strategy implies a need for frequent assessment of treatment response, such a remote monitoring approach holds promise to predict the need for urgent clinic visits in patients with high disease activity, but also to reduce the number of clinical visits for well-controlled patients [12]. By consequence, remote monitoring of symptoms through mHealth-applications could present a possible cost-saving measure [13], even though the potential impact on quality of care warrants further investigation.

Most patients with rheumatic diseases seem to consider that an mHealth-application could be beneficial to their health, although such an app should be needs-tailored and co-developed by healthcare professionals [14, 15]. Moreover, it appears crucial to patients for an mHealth-app to facilitate communication with their caregivers, to provide disease-specific information and to reinforce social support [16]. However, most available apps do not meet these criteria [17] and care models based on these applications have to date only sparsely been introduced in practice [18]. Moreover, previous studies have raised concerns about user disengagement with mHealth-applications, illustrating the need to study ideal app-content to optimise compliance [19]. Finally, the opinions of stakeholders other than patients, such as nurses and rheumatologists, have received little attention in mHealth-literature.

Therefore, perceptions of multiple stakeholders including both patients and healthcare providers on mHealth-applications need to be assessed ahead of the development of an integrated mHealth-app for routine rheumatology care. To this end, this study aimed to explore the expectations and opinions of mHealth-inexperienced patients, nurses and rheumatologists on the possible benefits, barriers and essential features of an ideal mHealth-application in the care for patients with RA.

## Methods

We conducted a cross-sectional qualitative study investigating stakeholder perceptions on mHealth-applications in RA care. Patients with RA, rheumatologists and rheumatology nurses were identified as stakeholders and included in semi-structured focus group interviews. A focus group method was specifically chosen to stimulate constructive discussion and to make optimal use of group dynamics [20]. The study protocol was approved by the University Hospitals Leuven Ethics Committee and all participants gave informed consent before participation.

### Study design and participant recruitment

Patients were invited for focus group interviews at the University Hospitals Leuven, Belgium, Rheumatology clinic or via representatives of RA patient associations, whom we contacted via email. This way, not only patients in follow-up at an academic hospital were included, but also patients in non-academic or private practice care. Practicing rheumatologists were invited to participate during local peer quality groups, which are mandatory for physician accreditation in Belgium. Rheumatology nurses were recruited through the Flemish working group on rheumatology nursing and the Belgian Health Professionals association. We conducted 5 focus group interviews in four different municipalities across the region of Flanders, Belgium, between January and March 2020. One additional focus group of rheumatologists was conducted via Zoom due to the Covid19-pandemic. Interviews were semi-structured, and all were coordinated by the same moderator (DDC) and two observers (RVM & MVDP).

Rheumatologists, rheumatology nurses and two patient research partners were involved in the construction of the interview guide (Supplement 1). The interviews were initiated with several exploratory open-ended queries, inquiring about stakeholders’ initial perceptions about mHealth-applications, before going into more specific follow-up questions relating to possible benefits, barriers and recommended features of an mHealth-application for RA. During the focus groups, notes were taken by the observers to record participant body language and behavioural cues. All focus groups were audio-recorded, transcribed verbatim and anonymised for analysis.

### Analysis

Two independent investigators (MD & DDC) coded and analysed the transcripts in accordance with the methodology outlined in the Qualitative Analysis Guide of Leuven (QUAGOL) [21] and guided by an experienced researcher (RW). Three patient research partners validated the final results of this analysis.

According to the principles of grounded theory underlying the QUAGOL-method, the initial codes were grouped into a conceptual framework of themes (Supplement 2 and 3), that was subsequently confirmed alongside all individual transcripts. This framework was then deconstructed into perceived benefits and barriers for the implementation of mHealth-applications in the care for patients with RA. Additionally, recommended features of an mHealth-application according to all stakeholders were grouped into a separate framework. In order to identify features that contribute to user engagement, this framework was iteratively compared with the existing literature on persuasive principles. Persuasive principles are design techniques that motivate users of a system, such as an app, to adhere to a specific target behaviour, such as continued app-use [22]. In mHealth-literature, ample experience exists with the Persuasive Systems Design (PSD) model by Oinas-Kukkonen and Harjumaa [22–26], which is one of the different theories based on persuasive principles.

### Persuasive Systems Design (PSD)

The PSD-model proposes 28 persuasive design principles divided into 4 categories: Primary Task Support, Dialogue Support, Social Support and System Credibility. Primary Task Support constitutes all persuasive principles intended to support the user in carrying out their primary task, including the reduction of complex behaviour into simpler tasks. Dialogue Support refers to principles that provide a degree of feedback to the user, including praise, rewards and reminders. Social Support includes all principles that attempt to motivate users by leveraging social influence. Finally, System Credibility principles, including trustworthiness and a sense of authority, aim to increase the persuasiveness of a system by making it more credible.

## Results

In total, 2 focus groups consisting of rheumatology nurses (*n* = 16), 2 of patients (*n* = 17) and 2 of rheumatologists (*n* = 25) were conducted. None of the stakeholders were experienced in the use of mHealth-applications. Expected benefits and barriers for the implementation of mHealth-applications in the care for patients with RA were identified across all stakeholders and grouped into the following 6 overarching themes: efficiency of care, enabling patient empowerment, the burden of chronic app-use, motivational aspects, target group aspects and legal and organisational requirements (Table 1). Additionally, recommended features of an mHealth-application for RA were grouped according to the PSD-framework (Table 2).

**Table 1:** Expected benefits and barriers of mHealth-apps for RA, according to mHealth-inexperienced patients, nurses and rheumatologists

BENEFITS	BARRIERS
**Efficiency of care**	**The burden of chronic app-use**
Efficiency of care organisation (P)	Negative illness behaviour (P, N, R)
Efficiency of patient-caregiver interaction (N, R)	Chronicity (P)
	**Motivational aspects**
	Compliance and attrition (P, N, R)
	A tsunami of mHealth-apps (R)
	Need for instruction and guidance (N)
**Enabling patient empowerment**	**Target group aspects**
Visualising disease variability (P, N, R)	Lack of symptoms (P, N, R)
Patient-centred goals (R)	Symptom recognition (P, N)
Improving illness coherence (P, N, R)	Accessibility and acceptability (P, N, R)
Trust in care (P)	Evolving disease characteristics (R)
	**Legal and organisational requirements**
	Privacy concerns (N, R)
	Workload and financial compensation (R)

*P* patients, *N* rheumatology nurses, *R* rheumatologists

**Table 2:** Recommended features of mHealth-applications for RA according to stakeholders, grouped into categories of the PSD-model

	Patients	Nurses	Rheumatologists
**Primary Task Support**			
Data reduction	**♦**	**♦**	**♦**
Efficient data entry	**♦**	**♦**	**♦**
Automated processes			**♦**
Personalised frequency of data entry	**♦**		**♦**
Graphical or visual feedback of evolution	**♦**	**♦**	**♦**
**Dialogue Support**			
Reminders for data entry	**♦**	**♦**	**♦**
Alerts in case of red flags	**♦**	**♦**	**♦**
Suggestions of action in case of red flags	**♦**		
**Social Support**			
Direct communication with healthcare providers	**♦**		
Free-text box	**♦**		
**System Credibility**			
Integration with electronic medical patient files		**♦**	**♦**
Link with interventions		**♦**	**♦**
Monitoring of medication adherence		**♦**	**♦**
Purpose and relevance of data entry	**♦**	**♦**	**♦**
Valid measurement instruments		**♦**	**♦**

*PSD* Persuasive Systems Design

### Benefits

#### Efficiency of care

##### Efficiency of care organisation

Patients considered that implementing mHealth-applications into clinical practice might reduce the number of necessary visits to the rheumatology clinic. For instance, some patients expected an mHealth-application could be used as a decision-support tool to help rheumatologists prioritise patients in need of more urgent attention.

*“Knowing that some patients have to wait a long time for an appointment, while we are only there to say we’re doing well… Maybe this could help to make way for these other people.” (Pt 4, patient group 1)*.

##### Efficiency of patient-caregiver interaction

In addition, both nurses and rheumatologists emphasised the benefits such applications could provide for efficient communication with their patients. Specifically, all caregivers agreed that being able to review data collected in between clinic visits, together with their patients, could improve the clarity of their interactions.

*“(With an overview of the data) you could refer to a flare the patient seems to have had 2 months ago, and the patient can then respond, ‘yes, that was the case because of this…’.” (R 9, rheumatologist group 2)*.

Rheumatologists and nurses both expected this to save valuable time during consultations, again improving efficiency. However, caregivers strongly emphasised that an mHealth-application should never replace genuine social interaction.

#### Enabling patient empowerment

##### Visualising disease variability

Patients, nurses as well as rheumatologists considered the potential of an mHealth-application to record symptoms or measures of disease activity in between clinic visits as a crucial benefit. All stakeholder groups agreed that these additional insights into the variability of a patient’s disease could improve the quality of care for patients with RA.

*“When I visit the rheumatologist on a day that I’m feeling fine, I’ve already forgotten how bad I felt 2 weeks before.” (Pt 1, patient group 2)*.

##### Patient-centred goals

Moreover, by being able to look at symptoms or physical data submitted by a patient during and immediately related to their daily life activities, rather than retrospectively discussing them, rheumatologists expected to gain a more comprehensive picture of what is really important to their patient. This way, they considered that an mHealth-application could help to clarify particular personal goals that patients might be reluctant to discuss with their physician during a consultation.

*“To emphasise the goals of our patients that are rarely discussed during a consultation. The landmarks that a patient might aspire to: being able to play with their grandchildren, or play tennis even if they’re 82 years old, etc.” (R 1, rheumatologist group 1)*.

##### Improving illness coherence

Just as rheumatologists believed mHealth-apps could improve their understanding of patients’ needs, it was also assumed that these applications could help patients to better understand themselves. For instance, the ability to confront patients with symptoms they had previously reported was perceived by caregivers as a means of improving patients’ illness coherence.

*“I think it could help to teach patients to look at their disease in the right way.” (N 2, nurse group 2)*.

In addition, patients strongly believed that the provision of personalised education through an mHealth-application could benefit their understanding of the disease, a sentiment that was confirmed by both nurses and rheumatologists.

##### Trust in care

Some patients expressed that having access to an mHealth-application would make them feel safer, since it provides more information and a more comprehensive picture of their disease course to caregivers.

*“What if something’s wrong and you don’t know for sure? Well, this way you know, because your nurse and your rheumatologist are always informed.” (Pt 3, patient group 2)*.

### Barriers

#### The burden of chronic app-use

##### Negative illness behaviour

All stakeholder groups expressed concern that asking patients to frequently rate their symptoms with an mHealth-application could facilitate negative aspects of illness behaviour. For instance, both nurses and rheumatologists worried that patients paying excessive attention to their symptoms might aggravate the disease’s impact on their daily lives.

*“People shouldn’t be occupied with this every single day, because eventually your disease becomes the only thing you’re concerned about. After a while, you’ll become your disease.” (N 3, nurse group 2)*.

Patients voiced similar concerns and feared that an mHealth-application that requires them to rate their symptoms too frequently would leave them feeling deprived of their freedom or even “trapped” by their disease.

##### Chronicity

Some patients related these fears to the chronic nature of RA and worried that committing to an mHealth-guided follow-up strategy would therefore imply a life-long obligation.

#### Motivational aspects

##### Compliance and attrition

Patients, nurses and rheumatologists all expected attaining sufficient patient compliance with an mHealth-application to be a challenge. Moreover, all stakeholders expressed concerns that compliance with such an app would inevitably decrease over time.

*“When it starts to become a drag, you’ll stop using it.” (Pt 7, patient group 2)*.

Additionally, nurses were particularly concerned that some patients, when prompted to remotely report disease symptoms through an app, would tend to over- or understate the severity of their symptoms in an attempt to prompt or avoid clinic visits, respectively.

##### A tsunami of mHealth-apps

Rheumatologists indicated that the recent emergence of a large number of mHealth-applications might hamper patients’ inclination to use a particular app of interest.

##### Need for instruction and guidance

Nurses emphasised that time and effort would need to be invested to teach patients how to use an mHealth-application.

#### Target group aspects

##### Lack of symptoms

Patients expected a lack of symptoms would hamper their motivation to register data into an mHealth-application. Therefore, most patients assumed the early or active stages of RA would be the ideal window to target with such an application, which was confirmed by both nurses and rheumatologists.

*“If you feel fine and there are no concerns, I suppose you won’t keep remembering to use the app.” (Pt 5, patient group 1)*.

##### Symptom recognition

Nonetheless, it was acknowledged by patients that remotely recording symptoms requires a certain amount of experience in recognising exactly which symptoms are disease-related, which is often more difficult in the early stages of disease.

*“I can understand that recognising the symptoms could be more difficult for patients who have only recently been diagnosed or even don’t have a definitive diagnosis yet.” (Pt 3, patient group 2)*.

Uniting both these views, some caregivers believed an mHealth-guided care strategy could be useful to both patients with early and more established RA, suggesting an evolution from a complementary role of mHealth-apps in early disease to a more autonomous role later on in the disease course.

*“I think it could serve more as an ‘extra’ in the early disease, and maybe later on, when things are a bit more stable, it could replace some aspects of care.” (N 3, nurse group 2)*.

##### Accessibility and acceptability

Access to digital technologies and internet connection, as well as having a certain digital literacy, was identified by all stakeholders as a barrier for mHealth-applications. In some focus groups, older age was proposed as an example of this. Moreover, nurses indicated that language barriers and cultural differences could pose an additional challenge.

##### Evolving disease characteristics

Rheumatologists were concerned that remote follow-up with an mHealth-application would not be able to capture comorbidities or even changes in the defining characteristics of a patient’s underlying disease, implying that, at some point in time, the app could be reflecting signs and symptoms of a different health problem.

*“If at some point the diagnosis changes and is no longer correctly reflected by the app, I think that could pose a major problem. What if the diagnosis of inflammatory arthritis is not correct, or the disease has evolved into something else?” (R 16, rheumatologist group 2)*.

#### Legal and organisational requirements

##### Privacy concerns

While patients did not express a reluctance to share their personal data in the context of an mHealth-application, both nurses and rheumatologists were particularly concerned about the security of patients’ health information.

*“I can imagine some patients would be afraid their data would become available to insurers. These patients might rate their symptoms differently, fearing certain consequences.” (N 6, nurse group 2)*.

In part relating to these privacy concerns, rheumatologists strongly emphasised the need for an mHealth-application to be developed by healthcare providers and patients, rather than by commercial enterprises. This way, rheumatologists expected to retain more control over the content of such an application and the data processed by it.

##### Workload and financial compensation

Some rheumatologists were concerned that an mHealth-application would significantly increase the amount of patient data to be processed, consequently increasing their workload, while these activities are not currently reimbursed. Therefore, they felt that a tailored legal framework would be needed, including an adaptation of the remuneration system, for mHealth-guided care to be feasible.

### Recommended features of an mHealth-application

Table 2 provides a summary of recommended features for an ideal mHealth-application based on the suggestions of patients with RA, rheumatology nurses and rheumatologists in this study. The recommended features were grouped according to the framework of the PSD-model.

#### Primary task support

Reducing the amount and frequency of data to be entered by patients was identified as a priority for all stakeholders. Furthermore, data entry should be a quick and efficient process, while a degree of automation in both entering and processing of the data was preferred by rheumatologists. Patients, nurses and rheumatologists all expected visual or graphical feedback of disease activity scores or symptoms over time to improve patients’ compliance to app-use. Finally, both patients and rheumatologists suggested to personalise the frequency and content of requested data entry according to a patient’s disease activity, for instance extending the interval of symptom registration for patients with a stable disease.

#### Dialogue support

All stakeholder groups preferred an mHealth-application to provide reminders when data entry is due. Moreover, patients indicated they would feel more comfortable with mHealth-guided follow-up if the application would send out an alert when the results indicate an increase in disease activity, for instance suggesting them to contact their healthcare provider.

#### Social support

While patients did not expect an mHealth-app to facilitate communication with their peers, a direct communication with healthcare providers was identified by patients as a priority for an mHealth-app. The option of a ‘free-text box’ to further specify certain symptoms was suggested as a means to promote this.

#### System credibility

Nurses and rheumatologists strongly emphasised the need for an mHealth-application to be based on validated measures of disease activity. However, the interpretation of remotely monitored disease activity measures was perceived as a challenge, due to the subjective nature of many of these measures. Furthermore, care providers considered an mHealth-application should be integrated with electronic medical patient files or even linked to specific interventions, including physical therapy programs or the monitoring of medication adherence. For patients, it was particularly important to be convinced that their data would actually be used for their benefit.

## Discussion

In this cross-sectional qualitative study, we investigated the perceptions and recommendations of ‘app-naïve’ stakeholders on mHealth-applications in the care for patients with RA.

All stakeholders agreed on some expected benefits mHealth-applications could provide to clinical practice. Firstly, both patients and healthcare providers were convinced that using mHealth-apps could save valuable time and improve the efficiency of ambulatory care. Additionally, patients acknowledged that remote monitoring of RA disease activity via an app could allow rheumatologists to prioritise the allocation of clinic visits to those patients who need it most. These findings are in line with results from a recently published study, which showed this strategy was acceptable to patients with established RA when demand exceeded capacity [27]. Secondly, all stakeholders in our study were confident that mHealth-apps could enable patient empowerment by providing patients with more insight into the evolution of disease activity, as well as by conferring personalised education and enhancing patients’ trust in their caregivers. Moreover, patients recognised that, by collecting information about their daily lives via an app, they could grant healthcare providers a more comprehensive picture of what matters most to them. As studies have shown, being able to discuss the results of remote monitoring with their physician is highly valued by patients as a contributor to patient-caregiver interaction [11, 28]. All these factors might contribute to improved illness coherence, a crucial step towards patient empowerment [29].

In spite of these benefits, our study highlights a number of important barriers for mHealth-applications to become an integral part of daily clinical care for RA patients, including the necessity of an appropriate legal framework. For instance, concerns were raised towards data privacy, and who would be able to access patient information. Clear information should thus be provided towards all stakeholders to ensure trustworthy data processing. Moreover, several expected barriers for the implementation of mHealth-applications were specifically related to remote symptom monitoring. First, rheumatologists were concerned mHealth-based symptom monitoring would increase their workload. Therefore, mHealth-applications should aim to streamline the existing responsibilities of healthcare providers, for instance by providing more data, and should not result in additional tasks. Second, patients, nurses and rheumatologists all expressed concerns that frequently assessing disease symptoms entails a risk of reinforcing negative illness behaviour. Patients also worried this would confront them more often with the chronic nature of their disease. Similarly, in a recent study, patients who were interviewed after testing a novel mHealth-app, indicated that the app reminded them too much of their disease, resulting in an internal resistance to use the app [30]. Third, all stakeholders in our study were concerned about using data perceived to be subjective and highly patient-specific, such as levels of pain and fatigue. All these expected barriers illustrate the need to carefully consider in what way and how intensively an optimal mHealth-app should track disease activity.

As a final expected barrier, our study participants identified compliance to app-use as a challenge and expected this to further decline over time. Indeed, although previous research on mHealth in RA has shown generally high compliance rates, a decline in app-compliance over time has been suggested [31–33]. Interestingly, most stakeholders in our study assumed compliance would be particularly low when the disease is well-controlled. While it was suggested that the early stages of disease would therefore present the ideal window for mHealth-guided symptom-tracking, our results also show that a degree of experience in recognising disease symptoms appears crucial. Strikingly, while recent studies on mHealth-interventions have been very heterogeneous in nature, only one study has to date investigated an mHealth-guided intervention in patients with early or active disease [12, 18]. Therefore, the ideal target population for an mHealth-application remains a matter of debate.

In addition to perceived benefits and barriers for the implementation of mHealth-apps, our study participants also identified a number of features the ideal mHealth-application should provide. To explore the expected impact of these features on user engagement, we divided proposed features into 4 categories according to the PSD-model [22]. Although it could be argued that improving user engagement should also depend on support and empowerment, rather than being limited to persuasive designs, this model nonetheless provides a useful framework to identify motivational app-features. A recent study based on the PSD-model concluded that most mHealth-apps for chronic arthritis would benefit from more emphasis on Social Support and Dialogue Support-techniques [23]. In contrast, most features recommended by stakeholders in our study were related to improving Primary Task Support and System Credibility, such as efficient data entry and a secure link with medical records. We should note that features related to these categories could have partly been prompted by specific interview questions, such as “do you think these applications could be used to monitor your disease?” Nevertheless, all stakeholders emphasised the need for an mHealth-application to facilitate communication between patients and care providers, illustrating that Social Support-features would indeed be highly valued by such an app’s intended primary users. In contrast, communication between patients themselves via mHealth-apps was not mentioned in our focus groups, even though peer mentoring has been shown to be important to patients [34, 35]. Similarly, not many existing mHealth-apps supply such a platform towards patient communication or a patient community [23, 36]. Reasons for this are unknown, although this could be due to privacy concerns or even due to system credibility. However, peer support might prove to be an important facilitator for app-use and should be explored more in future research.

Our study has some limitations. Firstly, participants were interviewed in focus groups. While focus groups are an efficient method to interactively explore participants’ perceptions, some participants might have felt less comfortable to speak openly than they would have in individual interviews. However, focus groups provide a setting that often allows for constructive reasoning among stakeholders, which was of particular importance to gather specific recommendations on app-content. Moreover, two separate focus groups were conducted for each stakeholder group and exhibited comparable results, illustrating the robustness of our findings. Secondly, detailed demographic characteristics of participants could not be provided, since the collection of these data was not included in the study protocol. However, our focus groups did include patients from both academic and private practice or non-academic care settings, and patients from various age groups. None of the included patients had a recently diagnosed RA. Moreover, specific efforts were made to include nurses and rheumatologists with varying levels of experience as well as different practice settings across several different regions. Finally, most of our study participants had little or no experience with mHealth-applications. These results should therefore not be interpreted as experience-based suggestions for improvement. However, this ‘app-naïve’ study population is also a major strength of this study. This way, the opinions, concerns and recommendations for an mHealth-application, as perceived by its intended users, could serve as a blueprint to develop the optimal mHealth-app. Moreover, by investigating the perceptions of rheumatologists and rheumatology nurses in addition to patients with RA, our study provides a more representative overview of what all its intended users expect an mHealth-application to offer. In addition, the robustness of our findings was improved by analysing the results with an interdisciplinary research team, guided by the QUAGOL. Finally, patient research partners were involved in every stage of this study, providing additional credibility to our results.

## Conclusions

This study investigated possible benefits, barriers, and recommendations for the implementation of mHealth-applications in the care for patients with RA, as perceived by mHealth-inexperienced patients, rheumatologists, and rheumatology nurses. Participants expected mHealth-apps to improve the efficiency of care and enable patient empowerment but were concerned that these apps could reinforce negative illness behaviour and assumed compliance would be a challenge. A number of app-features were recommended to overcome these challenges. Finally, while the ideal target audience for an mHealth-app remains to be further determined, a tailored legal and organisational framework should be in place for these applications to become a part of routine care for patients with RA.

## Supplementary Information

**Additional file 1: Supplement 1.** Interview guide

**Additional file 2.**

**Additional file 3.**

## Data Availability

The original transcripts (pseudonymised, in Dutch) used and analysed during the current study are available from the corresponding author on reasonable request.

## Abbreviations

RA: Rheumatoid arthritis
LDA: Low disease activity
T2T: Treat-to-target
DMARD: Disease-modifying antirheumatic drug
mHealth: Mobile health
WAT: Wearable activity tracker
PRO: Patient-reported outcome
QUAGOL: Qualitative Analysis Guide of Leuven
PSD: Persuasive Systems Design
