# Supplementary material for: The ideal mHealth-application for rheumatoid arthritis: qualitative findings from stakeholder focus groups
Source: BMC Musculoskelet Disord. 2021 Aug 30;22:746. doi: 10.1186/s12891-021-04624-8 (PMC8406841; doi:10.1186/s12891-021-04624-8)
Supplement: Supplementary file 1 — Additional file 1: Supplement 1. Interview guide [file 12891_2021_4624_MOESM1_ESM.docx]

**Supplement 1. Interview guide**

**A. Patient focus groups**

*Opening question:*Have you ever used mobile health applications or wearable devices before? What was your experience with these apps or devices? What do you use them for?

*Topic questions:*

- Do you think mobile health applications or wearables could be used to the benefit of your RA?
- If so, what should such an app certainly contain or be able to do, for you to want to use it?

*Additional questions in case the following topics do not come up:*

- Do you think these applications could be used to monitor your disease?
- Do you think these applications could be used to guide your treatment?

**B. Nurse focus groups**

*Opening question:*
To your knowledge, do your patients use mobile health applications or wearable devices? What are your experiences with patients using these apps or devices?

*Topic questions:*

- Do you think mobile health applications or wearables could be used to the benefit of your patients with RA?
- If so, what should such an app certainly contain or be able to do, for you to recommend it to your patients?

*Additional questions in case the following topics do not come up:*

- Do you think these applications could be used to monitor the disease?
- Do you think these applications could be used to guide treatment?

**C. Rheumatologist focus groups**

*Opening question:*
To your knowledge, do your patients use mobile health applications or wearable devices? What are your experiences with patients using these apps or devices?

*Topic questions:*

- Do you think mobile health applications or wearables could be used to the benefit of your patients with RA?
- If so, what should such an app certainly contain or be able to do, for you to recommend it to your patients?

*Additional questions in case the following topics do not come up:*

- Do you think these applications could be used to monitor the disease?
- Do you think these applications could be used to guide treatment?

**D. General remarks**

For each interview, additional questions will be prepared to:

- Further probe and extend the topic/answer
- Encourage participants to provide more information
- Maintain focus on the topic at hand
- Close down the topic

*Probing questions:*

- Could you tell us a bit more about this?
- Could you give an example?
- How do you see this in concrete terms?
- What do you mean exactly?
- Why or why not?

*Encouraging questions:*

- What is your opinion about this?
- Would you like to add anything?
- What is your view on this?
- Is the question clear?

*Maintaining focus:*

- Interesting comments. What do the other participants think about this?
- This is interesting to hear, but will lead us too far today.
- Let us continue to the next question.

*Closing questions:*

- Would you like to add some final thoughts?
