# Supplementary figures and images for: The ideal mHealth-application for rheumatoid arthritis: qualitative findings from stakeholder focus groups
Source: BMC Musculoskelet Disord. 2021 Aug 30;22:746. doi: 10.1186/s12891-021-04624-8 (PMC8406841; doi:10.1186/s12891-021-04624-8)

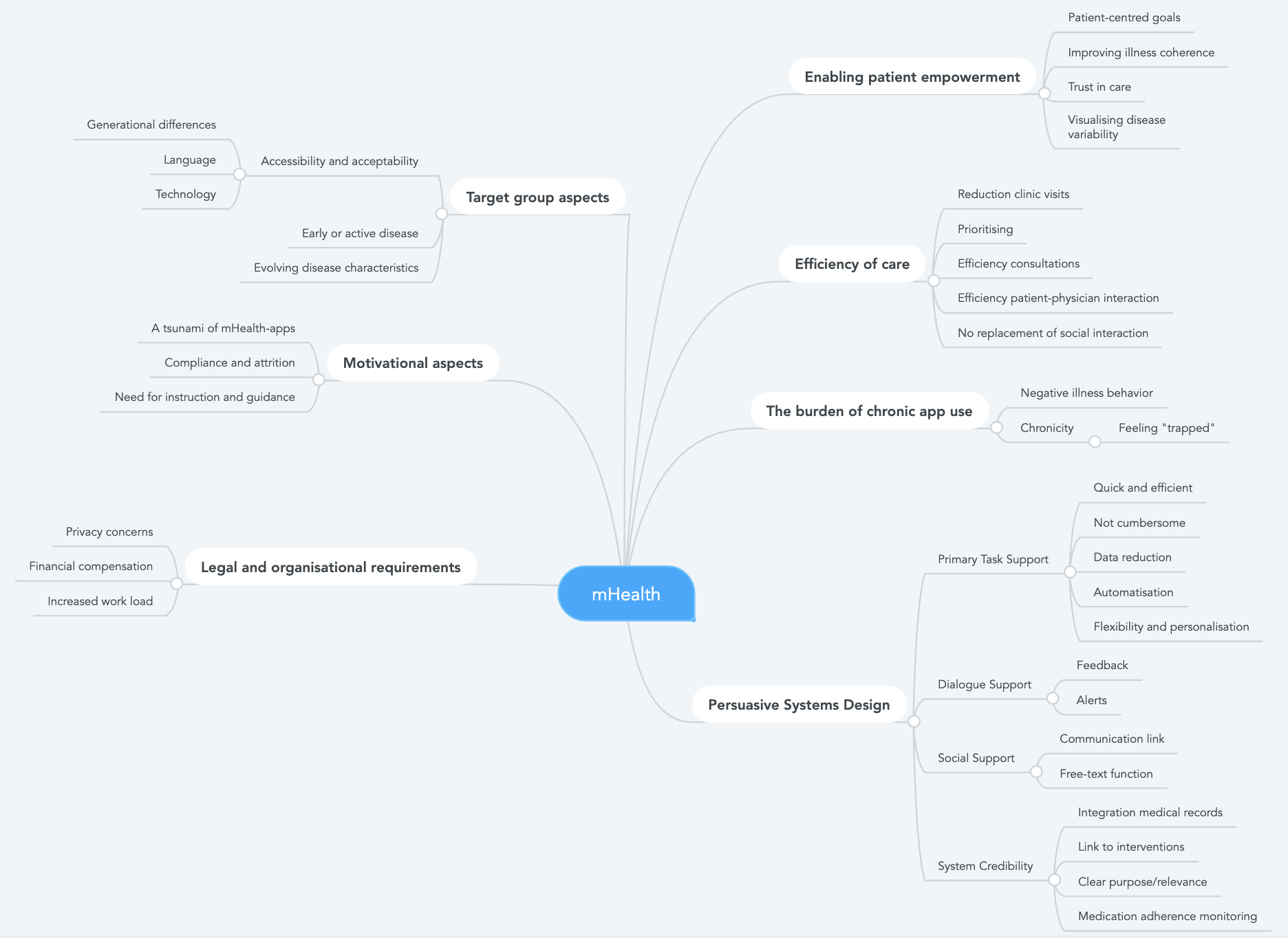

Supplement: Supplementary file 2 — Additional file 2. [file 12891_2021_4624_MOESM2_ESM.tiff]
