# Supplementary material for: The ideal mHealth-application for rheumatoid arthritis: qualitative findings from stakeholder focus groups
Source: BMC Musculoskelet Disord. 2021 Aug 30;22:746. doi: 10.1186/s12891-021-04624-8 (PMC8406841; doi:10.1186/s12891-021-04624-8)
Supplement: Supplementary file 3 — Additional file 3. [file 12891_2021_4624_MOESM3_ESM.docx]

**Efficiency of care**

- **Reduction clinic visits:** *“An advantage could be that you wouldn’t have to come into clinic for a while. Now I’m here every three months!” (Pt 1, patient group 1)*
- **Prioritising**: “*Knowing that some patients have to wait a long time for an appointment, while we are only there to say we’re doing well... Maybe this could help to make way for these other people.” (Pt 4, patient group 1)*
- **Efficiency consultations:** *“It would make it a lot easier if patients have already filled out information before the visit. That saves a lot of time.” (N 6, nurse group 1)*
- **Efficiency patient-physician interaction**: *“(With an overview of the data) you could refer to a flare the patient seems to have had 2 months ago, and the patient can then respond, ‘yes, that was the case because of this…’.” (R 9, rheumatologist group 2)*
- **No replacement of social interaction:**
  - *“Whether or not you can still pick up your child, for example, I don’t think you could put that into an app. Is anything then actually done with this information, does anyone listen to the patient’s story, like in real interaction?” (N 1, nurse group 1)*
  - *“You can monitor some things with an app, but the personal contact is still… Even by telephone, you always miss something. Even if it is just a look in someone’s eyes.” (R 9, rheumatologist group 2)*

**Enabling patient empowerment**

- **Visualising disease variability**: *“When I visit the rheumatologist on a day that I’m feeling fine, I’ve already forgotten how bad I felt 2 weeks before.” (Pt 1, patient group 2)*
- **Patient-centred goals**: *“To emphasise the goals of our patients that are rarely discussed during a consultation. The landmarks that a patient might aspire to: being able to play with their grandchildren, or play tennis even if they’re 82 years old, etc.” (R 1, rheumatologist group 1)*
- **Improving illness coherence**: *“I think it could help to teach patients to look at their disease in the right way.” (N 2, nurse group 2)*
- **Trust in care**: *“What if something’s wrong and you don’t know for sure? Well, this way you know, because your nurse and your rheumatologist are always informed.” (Pt 3, patient group 2)*

**The burden of chronic app use**

- **Negative illness behaviour**: *“People shouldn’t be occupied with this every single day, because eventually your disease becomes the only thing you’re concerned about. After a while, you’ll become your disease.” (N 3, nurse group 2)*
- **Chronicity / feeling “trapped”**:
  - *“Knowing that I would have to do this for the rest of my life, every week… I would eventually get tired of it, I think.” (Pt 5, patient group 1)*
  - *“Having to use this too often, you would lose some of your personal freedom.” (Pt 3, patient group 1)*

**Motivational aspects**

- **Compliance and attrition**: *“When it starts to become a drag, you’ll stop using it.” (Pt 7, patient group 2)*
- **A tsunami of mHealth-apps:** *“You often have 10 apps next to each other. You need one app that integrates what’s interesting for patients and what’s interesting for us. But if you have all of this separated, no way.” (R 1, rheumatologist group 2)*
- **Need for instruction and guidance:** *“People would need to be instructed as well. They would need to know when to report symptoms and when not to.” (N 1, nurse group 2)*

**Target group aspects**

- **Early or active disease:**
  - *“If you feel fine and there are no concerns, I suppose you won’t keep remembering to use the app.” (Pt 5, patient group 1)*
  - *“I can understand that recognising the symptoms could be more difficult for patients who have only recently been diagnosed or even don’t have a definitive diagnosis yet.” (Pt 3, patient group 2)*
  - *“I think it could serve more as an ‘extra’ in the early disease, and maybe later on, when things are a bit more stable, it could replace some aspects of care.” (N 3, nurse group 2)*
- **Accessibility and acceptability**:
  - Generational differences: *“That generation that is not really familiar with using a computer, they also get rheumatism. I don’t see them using tools like this.” (Pt 3, patient group 1)*
  - Language / Technology:
    - *“We regularly see patients who only speak Arabic, for example, or who don’t have a computer at home.” (N 3, nurse group 1)*
    - *“I still see about 20% of patients who just don’t have internet or a smartphone.” (R 2, rheumatologist group 2)*
- **Evolving disease characteristics**: *“If at some point the diagnosis changes and is no longer correctly reflected by the app, I think that could pose a major problem. What if the diagnosis of inflammatory arthritis is not correct, or the disease has evolved into something else?” (R 16, rheumatologist group 2)*

**Legal and organizational requirements**

- **Privacy concerns**: *“I can imagine some patients would be afraid their data would become available to insurers. These patients might rate their symptoms differently, fearing certain consequences.” (N 6, nurse group 2)*
- **Financial compensation:** *“Would there be a financial compensation for all the time invested in looking at data, telephone calls…?” (R 1, rheumatologist group 1)*
- **Increased workload:** *“It shouldn’t be the case that we receive all this information and continuously have to look at it.” (R 6, rheumatologist group 1)*
